# The involvement of microRNAs in neurodegenerative diseases

**DOI:** 10.3389/fncel.2013.00265

**Published:** 2013-12-19

**Authors:** Simona Maciotta, Mirella Meregalli, Yvan Torrente

**Affiliations:** ^1^Stem Cell Laboratory, Department of Pathophysiology and Transplantation, Centro Dino Ferrari, Università degli Studi di Milano, Fondazione IRCCS Cà Granda Ospedale Maggiore PoliclinicoMilan, Italy; ^2^Diabetes Research Institute, University of Miami Miller School of MedicineMiami, FL, USA

**Keywords:** microRNA, neurodegenerative diseases, biomarker, Parkinson's disease, Alzheimer's disease, amyotrophic lateral sclerosis, Huntington's disease

## Abstract

Neurodegenerative diseases (NDDs) originate from a loss of neurons in the central nervous system and are severely debilitating. The incidence of NDDs increases with age, and they are expected to become more common due to extended life expectancy. Because no cure is available, these diseases have become a major challenge in neurobiology. The increasing relevance of microRNAs (miRNAs) in biology has prompted investigation into their possible involvement in neurodegeneration in order to identify new therapeutic targets. The idea of using miRNAs as therapeutic targets is not far from realization, but important issues need to be addressed before moving into the clinics. Here, we review what is known about the involvement of miRNAs in the pathogenesis of NDDs. We also report the miRNA expression levels in peripheral tissues of patients affected by NDDs in order to evaluate their application as biomarkers of disease. Finally, discrepancies, innovations, and the effectiveness of collected data will be elucidated and discussed.

## Introduction

Neurodegenerative diseases (NDDs) are a family of disorders characterized by progressive loss of neuronal function and structure, resulting in neuronal death in the nervous system. Different types of NDDs exist, depending on the neuron population affected; the most common are Alzheimer's disease (AD), Parkinson's disease (PD), Huntington's disease (HD), and amyotrophic lateral sclerosis (ALS). A commonality of NDDs is that they are not monogenic or polygenic diseases, and they are even more complicated because several events take part in the pathogenesis independent of genetic mutations. The molecular events responsible for neurodegeneration include oxidative stress, axonal transport deficits, protein oligomerization and aggregation, calcium deregulation, mitochondrial dysfunction, neuron–glial interactions, neuroinflammation, DNA damage, and aberrant RNA processing. The greatest risk factor for neurodegeneration is advancing age in combination with mitochondrial DNA mutation and oxidative stress damage. Other possible causes include gender, poor education, endocrine conditions, oxidative stress, inflammation, stroke, hypertension, diabetes, smoking, head trauma, depression, infection, tumors, vitamin deficiencies, immune and metabolic conditions, and chemical exposure. Because the pathogenesis of many of these diseases remains unknown, the role of environmental factors needs to be considered.

In the last few decades, NDDs have become a major challenge in neurobiology due to their enormous and growing social and economic implications in society. For the same reason, increasing research efforts have investigated the underlying molecular mechanisms in order to find a cure. Based on the latest evidence reviewed here, miRNA deregulation is emerging as a contributor to neurodegeneration by influencing most of the mechanisms responsible for NDDs. Neurodegeneration can also be considered to be an RNA disorder (Johnson et al., 2012) in which microRNAs play a major role. Studying miRNA involvement in NDDs might also provide targets for innovative therapies. Until now, patients affected by NDDs have been surgically and pharmacologically treated without obtaining a resolute therapy, which is due primarily to the fact that therapeutic approaches for NDDs require the modulation of multiple targets and molecular pathways because they are multigenic diseases. Based on the evidence that a single miRNA can influence several target genes, a whole disease phenotype could potentially be modified by modulating a single miRNA molecule, which makes these RNA molecules very intriguing from a therapeutic point of view. Furthermore, the identification of deregulated miRNAs in patients affected by NDDs or any other disease might allow earlier diagnosis and the monitoring of disease progress. The main challenge of using proteins as targets for routine diagnostics is low sensitivity, reproducibility, and specificity (Johnson et al., 2012). In conclusion, the aim of this review is to elucidate the broad implications of miRNAs in NDDs, but also to point out the need to overcome technical difficulties related to the study of miRNAs in NDDs. Finally, we also report in detail what has been discovered thus far regarding the involvement of miRNAs in different NDDs in order to evaluate their potential as therapeutic targets.

## Non-coding RNAs

The sequencing of the human genome has demonstrated that the transcriptional output of the human genome is extremely rich in non-coding RNAs (ncRNAs) (Lipovich et al., 2010). Since this discovery, expectations regarding ncRNAs have increased exponentially. More importantly, the expectations have been supported by the development of next-generation sequencing technologies, which have revealed thousands of unknown ncRNAs. The vocabulary for ncRNAs is still far from saturated. The fascination of functional non-protein coding RNAs is that they represent a means for an organism's cells (cells that are genetically identical) to develop unique identities and functions. RNA is part of a mechanism that exerts control over DNA to guarantee the expression of a specific repertoire of genes at the appropriate level and with the appropriate timing. Two important classes of functional RNAs can be distinguished: long non-coding RNAs (lncRNAs) and small RNAs. LncRNAs account for the majority of transcription, they have no unifying structure or function, and they are solely defined as RNA transcripts greater than 200 nucleotides in length with no coding potential (Ponting et al., 2009). Relatively few lncRNAs have been characterized functionally, but increasing evidence suggests important roles for the thousands of uncharacterized transcripts. LncRNAs have been shown to target proteins to specific genomic loci, affecting transcription patterns (Plath et al., 2003; Silva et al., 2003; Kohlmaier et al., 2004; Zhao et al., 2008); to modulate the activity of protein-binding partners (Dreyfuss et al., 2002; Allen et al., 2004; Espinoza et al., 2004; Feng et al., 2006; Shamovsky et al., 2006; Mariner et al., 2008); to function as precursors for small RNAs (Kapranov et al., 2007; Fejes-Toth et al., 2009) to affect the processing of other RNAs (Hellwig and Bass, 2008); and to modulate translation, DNA methylation, and chromatin.

In contrast to lncRNAs, the biogenesis and function of small RNAs is well known and can be divided into five classes: (i) short interfering (si) RNAs (Elbashir et al., 2001a), (ii) small temporal (st) RNAs (Pasquinelli et al., 2000), (iii) heterochromatic siRNAs (Reinhart and Bartel, 2002), (iv) tiny non-coding RNAs (Ambros et al., 2003), and (v) micro (mi) RNAs (Lagos-Quintana et al., 2001; Lau et al., 2001; Lee et al., 2001). These small RNAs are processed from longer precursors and loaded into an Argonaute (Ago) family member within a large effector protein complex. The typical function of small RNAs is to mediate the post-transcriptional gene silencing (PTGS) of target RNA transcripts. The best understood class of small RNAs is miRNAs, which were first discovered by Lee et al. (1993). miRNAs are 21–22nt single-stranded RNA molecules that inhibit gene expression by binding to a complementary sequence in the 3'UTR of target genes (Bartel, 2004). These molecules originate from longer transcripts (pri-miRNA) that are processed by Drosha nuclease to yield a short hairpin “pre-miRNA,” which is then processed by Dicer to generate a double-stranded RNA of 21–22nt. Only one of the two strands is loaded into the RNA-induced silencing complex (RISC) that identifies target mRNA based on sequence complementarity with the miRNA. One of the core components of RISC is member of the Argonaute (Ago) protein family, in particular Ago1 and Ago2. After association with RISC, the choice of post-transcriptional repression is determined by sequence complementarity of the miRNA with its binding sequence on the 3′UTR of target mRNA: mRNA cleavage will happen when there is sufficient complementarity, otherwise inhibition of protein translation will occur. (Hammond et al., 2000; Elbashir et al., 2001a,b; Nykanen et al., 2001; Martinez et al., 2002; Schwarz et al., 2002). The relevance of miRNAs has increased with time; they are currently known to be involved in almost all biological processes and developmental programs (Bartel and Bartel, 2003; Carrington and Ambros, 2003; Hunter and Poethig, 2003). The first evidence that ncRNAs play a key role in neurodevelopment is the widespread transcription of ncRNAs in the developing mammalian brain (Lagos-Quintana et al., 2002; Krichevsky et al., 2003; Sempere et al., 2004; Smirnova et al., 2005; Bak et al., 2008). Next generation sequencing allowed the identification of a group of miRNAs that are enriched in the brain and whose expression varies according to area of the brain (Landgraf et al., 2007). In particular, neuronal-specific miRNAs have been demonstrated to control neuronal differentiation, excitability, and function. These brain-enriched miRNAs play a role in a wide range of neurodegenerative pathologies as disease-causing genes, biomarkers, or actors in pathogenesis. The idea of using miRNAs as therapeutic targets is not far from being realized. Two miRNA-based therapeutic approaches can be applied: miRNA mimics and anti-miRNAs. miRNA mimics are small RNA molecules with the same sequence as the mature miRNA of interest that are used to down-regulate the expression of target proteins mimicking the miRNA of interest. The desired effect is over-expression of miRNAs and down-regulation of their target mRNA, which can be used as a protective therapeutic strategy. This strategy has some important challenges that need to be overcome before moving into the clinic. First, the possibility exists that many out off object proteins might also be down-regulated because they are targets of the miRNA of interest. Second, the half-lives of mimics *in vivo* are not well known. Third, treating the brain with miRNA mimics is difficult because they need to pass through the blood-brain barrier (BBB). The second approach is to deliver RNA molecules with a sequence complementary to the miRNA of interest. Stoffel's group designed “antagomirs,” RNA snippets conjugated to cholesterol molecules that help the RNA enter a cell (Krutzfeldt et al., 2005). The limit of antagomirs as a possible tool for treating NDDs is that they are not able to cross the BBB and require a local injection. Another strategy to inhibit endogenous miRNAs is to deliver synthetic sponge mRNA, which contains several complementary binding sites for the miRNA of interest (Kluiver et al., 2012a,b). Certain long ncRNAs are able to base-pair with small RNAs, inhibiting the ability of miRNAs to bind to their targets. Therefore, lncRNAs are analogous to how artificial miRNA sponges function (Ebert et al., 2007). This hypothesis was demonstrated by Franco-Zorrilla et al. (2007) with the long ncRNA induced by phosphate starvation 1 (IPS1) in *Arabidopsis thaliana* (Catarecha et al., 2007). Future prospects regarding the administration of miRNAs as therapeutics for NDDs will be discussed later.

## Neurodegenerative diseases and miRNAs

### miRNAs in Parkinson's disease (PD)

PD is the second most common NDD, estimated to occur in approximately 1% of individuals >60 years of age, with 4.1–4.6 million people affected worldwide. PD is a progressive neurodegenerative disorder characterized clinically by bradykinesia, tremor, rigidity, and eventually postural instability (Shtilbans and Henchcliffe, 2012). These symptoms are attributed to a loss of dopaminergic neurons of the substantia nigra. The pathology spreads to involve other brain regions, including the amygdala, cingulate gyrus, and higher cortical regions, resulting in the development of dementia and psychosis. The disease itself is quite heterogeneous, and symptom progression is variable (Mouradian, 2012).

Despite rigorous research efforts, patient management and clinical research are still hampered by suboptimal methods for diagnosis, refining the prognosis, predicting individual responses to therapeutic interventions, and tracking disease progression. The critical reliance of dopaminergic neurons on a functioning miRNA network has been demonstrated in both cultured cells and *in vivo* (Kim et al., 2007). The miRNA machinery is important in NDDs in general and in PD in particular because the recognition of the amount of certain pathogenic proteins in specific neuronal populations is critical for the survival of neurons involved in the pathogenesis of disease. No cure is currently available for PD, and ongoing therapies are only directed at treating the most bothersome symptoms. Treatment approaches include medication (dopaminergic administration) and surgical therapy. Other strategies include general lifestyle modifications (rest and exercise), physical therapy, support groups, occupational therapy, and speech therapy. Nevertheless, new experimental therapies are under investigation and ongoing clinical trials are testing the efficacy of anti-inflammatory (pioglitazone) and parasympathomimetic (rivastigmine) drugs, ganglioside administration, and stemcell-based therapies. Even though PD is a multigenic disease, one of the most promising therapeutic approaches is to compensate biologically for the genetic defects responsible for PD pathogenesis. Some efforts have been made in this direction in the field of miRNAs, and the results are encouraging, even if far from clinical implementation.

#### miR-7/miR-153 regulation of α-synuclein

A negative correlation has been reported with specific miRNAs for two of the genes involved in PD: α-synuclein (SNCA) and leucine-rich repeat kinase2 (LRRK2). SNCA localizes in presynaptic terminals, where it associates with the plasma membrane. The protein is widely expressed in the adult brain, particularly the neocortex, hippocampus, and substantia nigra (Jakes et al., 1994; Mori et al., 2002; Wislet-Gendebien et al., 2008). The 3′UTR of the human protein is more than twice as long as the coding sequence and highly conserved (Sotiriou et al., 2009). This reports simply a relevant role for the 3′in stabilizing SNCA mRNAs and regulating its translation into protein. Point mutations and gene duplication and triplication events in the SNCA locus have been identified in a number of families with autosomal dominant early onset PD (Singleton et al., 2003; Wood-Kaczmar et al., 2006). Higher expression of wild-type SNCA and expression of the three mutant forms of SNCA give rise to insoluble aggregates that constitute the main structure of the Lewy Bodies (Masliah et al., 2000; Tan and Skipper, 2007; Saiki et al., 2011). Thus, down-regulation of SNCA represents a possible mechanism for resolving PD. Two miRNAs have been demonstrated to inhibit the expression of SNCA: miR-7 and miR-153 (Junn et al., 2009; Doxakis, 2010) (Table 1). Both miRNAs are highly enriched in the brain (Bak et al., 2008), and their sequences are conserved among different organisms. miR-153, in particular, is conserved across vertebrate species. Both miRNAs inhibit SNCA mRNA and protein (Junn et al., 2009; Doxakis, 2010), with an additive effect (Doxakis, 2010). Interestingly, the expression profile of these two miRNAs in the brain of post-natal day 1 mice is similar to α-synuclein protein and mRNA, and has been localized primarily to the neurons of the midbrain, hippocampus, and cortex (Junn et al., 2009; Doxakis, 2010). Co-localization of a miRNA with its target gene suggests tight control of the amount of the target gene produced.

**Table 1:** **Specific target genes of miRNAs involved in neurodegeneration are listed**.

**NDDs**	**miRNAs**	**Target genes**	**References**
PD	miR-7	DP	Junn et al., 2009; Doxakis, 2010
	miR-153	E2F1	Doxakis, 2010
	let-7	LRRK2	Junn et al., 2009; Gehrke et al., 2010
	miR-184^*^	LRRK2	Gehrke et al., 2010
	miR-433	FGF20	Davis et al., 2005; Wang et al., 2008a,b
	miR-205	LRRK2	Cho et al., 2013
AD	miR-106a, -520c	APP	Patel et al., 2008
	miR-20a, -106a/b, -17	APP	Hebert and De Strooper, 2009
	miR-16, -101	APP	Long and Lahiri, 2011
	miR-147, -655, -323-3p, -644, -153	APP	Delay et al., 2011
	miR-124	APP splicing	Smith et al., 2011
	miR-29a, -29b-1, -9	BACE1	Hebert and De Strooper, 2009
	miR-298, -328, -195	BACE1	Boissonneault et al., 2009; Zhang, 2012
	miR-124	BACE1	Fang et al., 2012
	miR-98	IGF1	Hu et al., 2013
	miR-181c, -137	SPTLC1	Geekiyanage and Chan, 2011
	miR-29a, -29b-1, -9	SPTLC2	Geekiyanage and Chan, 2011
	miR-34a	TAU	Dickson et al., 2013
ALS	miR-206	HDAC4	Williams et al., 2009
	miR-9	NEFH	Haramati et al., 2010
HD	miR-9	REST	Juliano et al., 2008
	miR-9^*^	CoREST	Juliano et al., 2008
	miR-196a	mut-Htt	Cheng et al., 2013

*NDDs, neurodegenerative diseases; PD, Parkinson's Disease; AD, Alzheimer Disease; ALS, Amiotrophic Lateral Sclerosis; HD, Huntington's disease*.

#### miR-205/let-7/miR-184^*^ regulation of LRRK2

LRRK2 is a member of the leucine-rich repeat kinase family and is present largely in the cytoplasm, but also associates with the mitochondrial outer membrane. It is highly expressed in the brain, with the highest levels of expression in the hippocampus and striatum (Galter et al., 2006; Melrose et al., 2006). LRRK2 is involved in the early development of neuronal processes (Parisiadou et al., 2009) and gain-of-function mutations cause familial as well as sporadic PD (Zimprich et al., 2004). Recent investigations in flies have demonstrated that the mutated form of LRRK2 (mut-LRRK2) is responsible for a reduced miRNA-mediated gene repression. This is due to the fact that mut-LRKK2 physically interacts with Ago1 and Ago2—two components of the RISC—inducing their down-regulation in aged Drosophila Melanogaster (Gehrke et al., 2010). Gehrke et al. also investigated the possible target mRNAs whose translation is induced by mut-LRRK2 and identified E2F1 and DP. Flies expressing mut-LRRK2 were in fact characterized by higher expression levels of E2F1 and DP, and down-regulation of E2F1 and DP suppressed the death of dopaminergic neurons. Finally Gehrke S et al. demonstrated that miR-184^*^ and let-7, respectively, repressed E2F1 and DP (Table 1) and that inhibition of these miRNAs in wild-type animals was sufficient to phenocopy pathogenic LRRK2. In line with this, both let-7 and miR-184^*^ have been demonstrated to regulate dopaminergic survival and activity (Junn et al., 2009; Gehrke et al., 2010). Regardless the role of mut-LRRK2 in PD, latest studies investigated the consequences of wild-type LRRK2 deregulation in PD pathogenesis. In particular LRRK2 gene locus was identified as a genetic risk factor for the more common sporadic PD (Satake et al., 2009; Simon-Sanchez et al., 2009), indicating that alteration of its expression might be part of PD etiology. Moreover, up-regulation of LRKK2 in an animal model of PD quickened neurodegeneration (Lin et al., 2009). Basing on these evidences, Cho et al. analyzed the expression levels of LRKK2 (protein and mRNA) in the frontal cortex tissue of 8 sporadic PD patients and relative control subjects. No differences in the mRNA levels were found but affected brains were characterized by higher expression levels of LRRK2 protein, suggesting a miRNA-mediated regulation of this protein. *In silico* analysis has demonstrated a predicted binding site for miR-205 in the 3′UTR of LRKK2 and *in vitro* experiments confirmed a direct inhibition of LRKK2 via miR-205. Finally they demonstrated that transfection of miR-205 in the neurons expressing a PD-related LRKK2 R1441G mutant prevented the neurite outgrowth defects (Cho et al., 2013).

#### miR-433 regulation of FGF20

Fibroblast growth factor 20 (FGF20) is a neurotrophic factor preferentially expressed in the substantia nigra that sustains the survival of dopaminergic neurons (Ohmachi et al., 2000, 2003). In contrast to this pro-survival activity, FGF20 treatment of human neuroblastoma cell line SH-SY5Y increases the amount of endogenous SNCA, demonstrating an anti-survival role of FGF20 in dopaminergic neurons. Single nucleotide polymorphisms (SNPs) in the 3′UTR of this gene (i.e., rs1721100, ss20399075, and rs12720208) have been found to be associated with PD (Wang et al., 2008a). Importantly, the latest polymorphism is within the miR-433 binding site (Davis et al., 2005), which is highly enriched in the brain. Wang et al. demonstrated that SNP rs12720208 avoids inhibition by FGF20 through miR-433 (Wang et al., 2008a). Finally, subsequent investigations failed to confirm a relationship between the rs12720208 genotype, FGF20, and SNCA. These discrepancies are often related to the ethnic origins or genetic backgrounds of PD patients.

#### miRNAs in the peripheral tissues of PD patients

The use of biomarkers in PD is a moot point, and no reliable biomarker exists for this NDD, with the exception of the monogenetic form of PD. With the increasing relevance of miRNAs in NDDs, some efforts have been made to investigate the possibility of miRNAs as biomarkers. In particular, qRT-PCR analyses of peripheral blood isolated from eight untreated PD patients (NT) and eight control subjects (CTR) showed that the expression levels of three miRNAs (miR-1, miR-22^*^, and miR-29a) distinguish NT from CTR (Margis et al., 2011) (Table 2). A second study was based on qRT-PCR analyses of plasma obtained from 31 NT and 25 CTR (Cardo et al., 2013) and identified seven over-expressed miRNAs (miR-181c, miR-331-5p, miR-193a-3p, miR-196b, miR-454, miR-125a-3p, and miR-137) in NT (Table 2). Discrepancies may be attributed to intrinsic differences between the sample types (Table 2).

**Table 2:** **miRNAs deregulation in NDDs patients**.

**NDD**	**miRNAs**	**Source**	**Changes**	**Patients (P) and controls (C)**	**References**
PD	miR-133b	SNC	Down-regulation	3P, 5C	Kim et al., 2007
	miR-34b/c	SNC	Down-regulation	11P, 6C	Minones-Moyano et al., 2011
	miR-1, -22^*^, -29a	Peripheral blood	Down-regulation	8P, 8C	Margis et al., 2011
	miR-181c, -331-5p, -193a-p, -196b, -454, -125a-3p, -137	Plasma	Over-expression	31P,25 C	Cardo et al., 2013
AD	miR-34a, -181b	PBMC	Over-expression	16P, 16C	Schipper et al., 2007
	miR -26a, -27b, -30e-5p, -34a, -92, -125, -145, -200c, -381, -422a, -423	Hippocampus, cerebellum, medial frontal gyrus	Over-expression	15P, 12C	Cogswell et al., 2008
	miR-9, -132, -146b, -212		Down-regulation		
	let-7f, miR-105, -125a, -135a, -138, -141, -151, -186, -191, -197, -204, -205,- 216, -302b, -30a-5p, -30a-3p, -30b, -30c, -30d, -32, -345, -362, -371, -374, -375, -380-3p, -429, -448, -449, -494, -501, -517, -517b, -518b, 518f, 520a^*^, 526a	Cerebrospinal fluid	Over-expression	10P, 10C	
	miR-10a, -10b, -125, -126^*^, -127, 142-5p, -143, -146b, -154, -15b, -181a, -181c, -194, -195, -199a^*^, -214, -221, -328, -422b, -451, -455, -497, -99a		Down-regulation		
	miR-9, -125b, -146a, 155	Cerebrospinal fluid and brain tissue derived extracellular fluid	Over-expression	3P, 3C	Lukiw, 2007
	miR-26b	Substanzia nigra	Over-expression	10P, 8C	Absalon et al., 2013
ALS	miR-338-3p	Whole blood	Over-expression	12P, 8C	De Felice et al., 2012
	miR-451, -1275, -328, -638, -149, -665, -583		Down-regulation		
	miR-27a, -155, -146a, -32-3p	CD14^+^CD16^−^ monocytes	Over-expression	8P, 8C	Butovsky et al., 2012
	miR-146^*^, -524-5p, 582-3p	Spinal cord	Over-expression	5P, 3C	Campos-Melo et al., 2013
	miR-24-2^*^, -142-3p, -142-5p, -1461, -146b, -155	Spinal cord	Over-expression	16P, 12C	Koval et al., 2013
HD	miR-29a, -330	Brodmann's area 4	Over-expression		Johnson et al., 2008
	miR-132		Down-regulation		
	miR-132, -196, -486	Brodmann's area 4	Over-expression	19P, 7C	Packer et al., 2008
	miR-9, -9^*^, -124, - 29b, 17-3p, -22, -485-5p, 500, -222		Down-regulation	19P, 7C	
	miR-100, -151-3p,-16, -219-2-3p, -27b, -451, -92a	Frontal cortex and striatum	Over-expression	11P, 11C	Marti et al., 2010
	miR-128, -139-3p,-222,-382,-433,-483-3p	Frontal cortex and striatum	Down-regulation	11P, 11C	Marti et al., 2010
	miR-34b	Plasma	Over-regulation	27P, 12C	Gaughwin et al., 2011

*NDDs, neurodegenerative diseases; PD, Parkinson's Disease; AD, Alzheimer Disease; ALS, Amiotrophic Lateral Sclerosis; HD, Huntington's disease*.

### miRNAs in Alzheimer's disease (AD)

AD is the most common form of dementia in people over 65 years of age. The disease is characterized by progressive neuronal loss and inflammation affecting memory, language, behavior, and cognition. The disease is characterized by amyloid-β (Aβ) deposition, neurofibrillary tangle (NFT) formation, and extensive neuronal degeneration in the brain. Aβ is derived from the sequential cleavage of amyloid precursor protein (APP) by beta-site APP-cleaving enzyme 1 (BACE1) and the γ-secretase complex. The precise pathological mechanisms underlying AD are currently unknown. Clinical and research evidence indicates that aberrant regulation of miRNA-dependent gene expression is closely associated with molecular events responsible for Aβ production, NFT formation, and neurodegeneration (Hebert and De Strooper, 2007, 2009; Hebert et al., 2008; Wang et al., 2008b). The regulation of APP is complex but represents a great challenge in the treatment of AD patients. Current drug discovery approaches in AD have focused on (i) preventing Aβ formation or increasing “normal” APP processing through the inhibition of γ- and β-secretase or the activation of α-secretase activity (Palop and Mucke, 2010; Saido and Leissring, 2012; Schenk et al., 2012); removing existing amyloid deposits via immunotherapeutic approaches, e.g., antibodies or vaccines against amyloid (Schenk et al., 2012). The miRNA field has moved in the same direction, and miRNAs have been discovered to regulate APP expression in three different ways: directly, indirectly, and by regulating the alternative splicing of its mRNA.

#### Direct inhibition of APP via miRNAs

Direct regulation of APP is mediated by miRNA binding to a specific sequence in the 3'UTR. Several miRNAs that inhibit APP expression *in vitro* have been identified, including miR-106a and miR-520c; members of the miR-20a family (e.g., miR-20a, miR-106a/b, miR-17) (Hebert and De Strooper, 2009); miR-16 and miR-101 (Vilardo et al., 2010; Long and Lahiri, 2011); and miR-147, miR-655, miR-323-3p, miR-644, and miR-153 (Delay et al., 2011) (Table 1). Only a few of these miRNAs are deregulated in the brains of AD patients (Hebert et al., 2008; Nunez-Iglesias et al., 2010), and it is difficult to determine which of these miRNAs regulate APP *in vivo*.

#### Indirect inhibition of APP via miRNAs

Indirect inhibition of APP via miRNAs is through the direct down-regulation of genes in pathways regulating the expression, function, or processing of this protein. β-secretase BACE1, insulin-like growth factor 1 (IGF-1), and serine palmitoyltransferase (SPT) influence APP expression and are modulated by miRNAs.

BACE1 plays a pivotal role in regulating Aβ production by cleaving APP and releasing APPβ. Hebert et al. demonstrated the *in vitro* inhibition of BACE1 by miR-29a, miR-29b-1, and miR-9 and confirmed an association between the down-regulation of these miRNAs and AD (Hebert et al., 2008). Mice over-expressing miR-29c are characterized by the down-regulation of BACE1 levels, demonstrating an *in vivo* effect on BACE1 modulation (Zong et al., 2011). Other studies demonstrated a negative correlation between BACE1 and miR-298/miR-328/miR-195 in several animal models of AD and confirmed direct inhibition in different mouse cell lines (Boissonneault et al., 2009; Zhu et al., 2012). Finally, the most conserved and abundantly expressed nervous system-specific miR-124 has been shown to inhibit BACE1 expression in cultured rat PC12 cell lines and primary cultured hippocampal neurons, a cellular model of AD (Fang et al., 2012).

De-regulation of IGF-1-mediated signaling has been correlated with AD (Rosario, 2010). IGF-1 function in the brain includes Aβ clearance from the brain and phosphorylation of tau (Hong and Lee, 1997; Vargas et al., 2011). Hu et al. showed that the expression of miR-98 negatively correlates with the IGF-1 expression level in a mouse model of AD. Furthermore, over-expression of miR-98 in cellular models of AD is responsible for the down-regulation of IGF-1, enhanced Aβ production, and tau phosphorylation (Hu et al., 2013).

SPT, a heterodimer composed of serine palmitoyltransferase long chain 1 (SPTLC1) and serine palmitoyltransferase long chain 2 (SPTLC2), is the first rate-limiting enzyme in the de novo ceramide synthesis pathway (Hannun and Obeid, 2008). Membrane ceramides are known to contribute to AD pathology by facilitating the mislocation of BACE1 and γ-secretase to lipid rafts, thereby promoting Aβ formation (Lee et al., 1998; Vetrivel et al., 2005). Interestingly, SPT is increased in the brain of sporadic AD patients (Geekiyanage and Chan, 2011) with up-regulation of several miRNAs, including miR-137, miR-181c, miR-9, miR-29a, miR-29b-1, and miR-15. *In vitro* luciferase assay confirmed direct inhibition of SPTLC1 by miR-181c and miR-137 and of SPTLC2 by miR-29a, miR-29b1, and miR-9. Moreover, a negative correlation has been demonstrated between the expression levels of these miRNAs and their relative target genes, SPTLC1 and SPTLC2, in the frontal cortices of sporadic AD patients (Geekiyanage and Chan, 2011).

#### miRNAs regulating the alternative splicing of APP

Human APP exists as three major isoforms (APP751, APP770, and APP695) originating from alternative splicing. Isoforms APP751 and APP770 are widely expressed and contain the Kunitz protease inhibitor (KPI) domain encoded by exon7, but only APP770 contains the putative glycosylation domain OX2 encoded by exon8. The APP695 isoform is majorly expressed in neurons (Zhang et al., 2011) and contains neither the KPI nor OX2 domains. Changes in the expression profile of neuronal APP are associated with an increase in Aβ production (Donev et al., 2007). Higher expression of APP isoforms containing exons 7 and 8 is found in various areas of the brains of AD patients (Golde et al., 1990; Neve et al., 1990; Jacobsen et al., 1991; Tanzi et al., 1993; Rockenstein et al., 1995). To investigate the involvement of miRNAs in the regulation of APP splicing, Smith et al. created a forebrain-specific Dicer conditional knock-out mouse in which post-mitotic neurons were characterized as having increased levels of APP751 and APP770 isoforms. Because miR-124 plays a pivotal role in neuronal maintenance and splicing (Makeyev et al., 2007; Papagiannakopoulos and Kosik, 2009), Smith et al. induced the ectopic expression of miR-124 in Neuro2a cells, which was enough to induce the skipping of exons 7 and 8 by inhibiting polypyrimidine tract binding protein 1 (PTB1). In addition, and supporting this observation, lower expression of miR-124 was measured in the brains of AD patients (Smith et al., 2011).

#### miR-34a/miR-26b regulation of tau protein

The microtubule-associated protein tau promotes the assembly and stability of microtubules (Weingarten et al., 1975; Drubin and Kirschner, 1986). It is involved in many NDDs, collectively known as tauopathies (Lee et al., 2001). In the case of AD, tau is hyperphosphorylatated and accumulates in the cytoplasm where it gives origin to intraneuronal protein aggregates known as NFTs (Kosik et al., 1986; Nukina and Ihara, 1986; Wood et al., 1986). Although alterations in tau protein are not considered the earliest event in AD pathogenesis, reduction in its expression levels may be safe and beneficial to prevent or treat AD (Rapoport et al., 2002; Roberson et al., 2007; Ittner et al., 2010; Vossel et al., 2010). In this optics, Dickson et al. investigated the role of the 3′UTR of human tau mRNA in regulating tau expression. Using different prediction algorithms, they found several miRNA-binding sites and they were able to validate direct inhibition of human tau by miR-34a (Dickson et al., 2013). Another approach to inhibit NFT formation is represented by regulating the phosphorilation status of tau protein. Tau is in fact a phosphoprotein that contains more than 80 potential phosphorylation sites (Hanger and Noble, 2011). As mentioned above, hyperphosphorilation of tau causes insoluble aggregates into the cytoplasm of neurons. In regard, Absalon et al. identified a specific miRNA (miR-26b) that rises in the substantia nigra at early stages of AD (Braak III) and remains elevated in the pathological area of human AD brain during disease progression. A target mRNA of miR-26b was confirmed to be Retinoblastoma (Rb). Both over-expression of miR-26b and down-regulation of Rb in primary cortical neurons showed activation of cyclin-dependent kinase 5 (Cdk5) and enhanced tau phosphorylation, followed by apoptosis and neurodegeneration *in vitro* (Absalon et al., 2013). AntagomiR-26b based therapy might not only decrease tau phosphorylation and NTF formation, but also enhances neuronal survival.

#### miR-146 regulation of presenilin

As described by Haas et al. the APP undergoes successive proteolysis by β- and γ-secretases to produce the Aβ that characteristically deposits in AD brain (Hass et al., 2009). γ-Secretases is a large complex of four integral membrane proteins, with presenilin (PSEN) as the catalytic subunit. Dominant mutations in the genes encoding for presenilins (PSEN1 and PSEN2) are the most common cause of familial early-onset Alzheimer's disease (Brouwers et al., 2008). These mutations alter the biochemical character of the γ-secretase complex and its interaction with the APP substrate, so that a longer and aggregation-prone form of Aβ is produced (Mucke and Selkoe, 2011). It is also to note that presenilins function is likely to be relevant to the development of sporadic AD. For all above reasons, presenilins together with β- and γ-secretases are top targets for AD drug discovery. In addition to its role in Aβ generation, PSEN2 was demonstrated to modulate the microglia activity (Jayadev et al., 2013). More in detail, Jayadev et al. demonstrated that *in vivo* deficiency of PSEN2 associated with exaggerated pro-inflammatory state in microglia. Basing on this evidence, they hypothesized that presenilin disfunctions could contribute to AD neurotoxic inflammation (Jayadev et al., 2010). In order to elucidate the underlining molecular mechanisms, PSEN2 knockout (KO) and wt microglia were analyzed for differential miRNA expression. The expression profiles of several miRNAs involved in the regulation of innate immune signaling were perturbed in PSENKO microglia, including miR-146 that is a potent negative regulator of innate immunity. This observation suggested that PS2 modulates cytokine responses via inhibition of miR-146. In line with this evidence, the target mRNA of miR-146a IRAK-1 (interleukin-1 receptor-associated kinase-1) was increased in PS2KO microglia. One of the function of IRAK-1 is to be a mediator of IL-1 (interleukin-1) signaling (Cao et al., 1996) and a critical regulator of Toll-like receptor (TLR) signal transduction (Swantek et al., 2000). When activated, IRAK-1 binds to NFkB thereby promoting nuclear localization and transcriptional activity (Flannery and Bowie, 2010). Indeed PS2KO vs. wt. microglia showed increased NFkB activity upon stimulation with lipopolysaccharide (LPS). Jayadev et al. strongly demonstrated that PSEN2 influences microglia activity but the exact mechanism by which PSEN2 carries out this task via miR-146 modulation still need to be elucidated (Jayadev et al., 2013).

#### miRNA profile of the brain and peripheral tissues in AD

In most cases, AD can only be diagnosed by neuropsychological studies, neuroimaging, and clinical data from patients that allow characterization as probable or possible AD patients (Mckhann et al., 1984) with a sensitivity of 93% and specificity of 55%. Furthermore, diagnosis is far more difficult in early and unusual presentations of the disease. Several research efforts have examined miRNAs in order to identify potential biomarkers. In 2007, the first small-scale profiling of miRNAs was performed on the hippocampal region of fetal, adult, and AD brains (Lukiw, 2007). Since then, several large-scale analyses have been performed on different AD tissues, including brain, peripheral blood, and cerebrospinal fluid (CSF) (Schipper et al., 2007; Cogswell et al., 2008; Hebert et al., 2008; Wang et al., 2008a; Nunez-Iglesias et al., 2010; Shioya et al., 2010). Nevertheless, miRNA expression studies on AD patients have had either no or very little overlap in miRNA changes (Table 2). Schipper et al. analyzed blood mononuclear cells (BMC) from patients with sporadic AD using miRNA microarray analyses and found two miRNAs that are significantly up-regulated in AD subjects: miR-34a and miR-181b (Schipper et al., 2007) (Table 2). Cogswell et al. performed qRT-PCR analysis on brain tissue and CSF from AD patients, identifying a set of miRNAs, so-called AD-specific miRNAs, that are differentially expressed in the brain and altered in the CSF of AD patients (Cogswell et al., 2008) (Table 2). Finally, Lukiw et al. group recently characterized the miRNome of AD CSF and short post-mortem interval brain tissue-derived extracellular fluid (ECF) using fluorescent miRNA array, finding significant increases in miR-9, miR-125b, miR-146a, and miR-155 in AD CSF and ECF (Lukiw, 2007) (Table 2).

### miRNAs in amyotrophic lateral sclerosis (ALS)

ALS is often referred to as “Lou Gehrig's Disease.” It is a progressive, idiopathic, fatal NDD that affects nerve cells in the brain and spinal cord. Motor neuron loss gives rise to malfunctions in the muscle tissue, causing weakness, atrophy, and ultimately paralysis and death within 3 or 5 years of symptom onset. The disease occurs worldwide with an incidence of approximately 2 × 10^5^ and a prevalence of approximately 6–8 × 10^5^. Currently, there is only one FDA-approved compound; riluzole does not resolve the disease, but slows progression and extends survival with modest effects. The discovery of small molecules that change the course of disease in ALS is desirable. With the increasing relevance of miRNAs, many recent research efforts have investigated the role of these small RNA molecules in the pathogenesis of ALS. The data that have been obtained are encouraging but still in their infancy, as they demonstrated an involvement but are far from proposing a solution. Nevertheless, if we are able to improve our understanding of the pathogenesis of ALS, it could lead to the development of early and specific diagnostic methods and extend the life expectancy of ALS patients. No definitive diagnostic tests or biomarkers exist for ALS, and neurologists rely on clinical criteria for diagnosis. The development of novel biomarkers to evaluate disease progression could give us the ability to refine the design of therapeutic trials and reduce the costs of clinical trials (Kiernan et al., 2011).

#### miR-206 and re-innervation

One of the most promising studies toward an innovative approach to cure ALS was conducted by Williams et al. (2009). miRNAs are involved in the stress response in skeletal muscle (Van Rooij and Olson, 2007). Because ALS is characterized by paralysis of the lower limbs, Williams et al. investigated the miRNome of muscles isolated from the lower limbs of an animal model of ALS, SOD1 transgenic mice. MyomiR-206 (Chen et al., 2006; Rao et al., 2006) was strongly induced, and its up-regulation coincided with the onset of symptoms. After severing the sciatic nerve of wild-type mice to induce denervation of the lower leg muscles, higher expression levels of miR-206 were observed 10 days after surgery in fast-twitch muscles, suggesting the involvement of this miRNA in re-innervation. This hypothesis was confirmed when miR-206 was knocked out in SOD1 transgenic mice, demonstrating accelerated progression of ALS and shortened survival (Williams et al., 2009). The underlying molecular mechanism was investigated and miR-206 was found to induce the secretion of fibroblast growth factor binding protein 1 (FGFBP1) from muscle by inhibiting Histone deacetylase 4 (HDAC4) translation. FGFBP1 potentiates the effect of FGFs in the promotion of presynaptic differentiation at the neuromuscular junction (Fox et al., 2007).

#### miRNA biogenesis and ALS

Multiple studies have identified several dominant mutations in the 43-kDa trans-activating response region (TAR) DNA-binding protein (TDP-43) in both sporadic and familial ALS patients that are associated with other NDDs (Kabashi et al., 2008; Sreedharan et al., 2008; Pesiridis et al., 2009; Lagier-Tourenne et al., 2010). A functionally related gene, fused in sarcoma/translocation in liposarcoma (FUS/TLS), is also mutated in ALS (Kwiatkowski et al., 2009; Vance et al., 2009). These two DNA/RNA-binding proteins physically interact with one another and are physiologically involved in the regulation of RNA transcription and splicing (Giordana et al., 2010; Lagier-Tourenne et al., 2010). The exact mechanism by which these proteins become pathogenic in ALS remains uncertain, but the most assessed hypothesis is related to their nuclear/cytoplasmic imbalance (Kwiatkowski et al., 2009; Vance et al., 2009; Giordana et al., 2010). Moreover, Ling et al. discovered that ALS-associated forms of TDP-43 have longer half-lives, contributing to TDP-43 aggregation in ALS patients, and they have an increased affinity for FUS/TLS (Ling et al., 2010).

By combining tandem-affinity purification and quantitative mass-spectrometry analysis, Ling et al. discovered that TDP-43 is associated with multiple hnRNP proteins and the Drosha microprocessing complex (Ling et al., 2010). Similarly, data indicated that Drosha protein is a putative FUS interactor (Gregory et al., 2004). Association with Drosha and mislocation of TDP-43 and FUS/TLS suggests de-regulation of miRNA biogenesis in ALS. Independent from this study, knocking down TDP-43 in the human Hep-3B cell line was later shown to replicate the changes occurring in the total miRNA population (Buratti et al., 2010). A relationship was also demonstrated between TDP-43 and brain-enriched miR-9; loss of Drosophila TDP-43 was characterized by down-regulation of miR-9a and TDP-43 influenced sensory organ precursor (SOP) cells in Drosophila through miR-9a (Li et al., 2013). Regarding FUS/TLS, its down-regulation in neuroblastoma cell line SK-N-BE affected the biogenesis of a large class of miRNAs, including neuronal isoforms. FUS/TLS is recruited at the chromatin, where it directly binds pri-miRNAs, facilitating Drosha loading (Morlando et al., 2012).

#### miR-9 regulation of neurofilaments

Neurofilaments are components of the neuronal cytoskeleton and provide structural support to the axons. They are assembled from light, medium, and heavy subunits, creating three different types of neurofilaments: light (NEFL), medium (NEFM), and heavy (NEFH). If the expression of neurofilaments is not well orchestrated, axonal cytoskeletal defects occur (Julien, 1999; Liem and Messing, 2009). Perturbation of the fine neurofilaments is associated with the development of human ALS (Figlewicz et al., 1994; Tomkins et al., 1998; Al-Chalabi et al., 1999). The 3′UTRs of neurofilament-encoding genes appear to interact with an uncharacterized trans-acting factor that is attenuated in ALS (Haramati et al., 2010), which might be miRNAs. In support of this hypothesis, ablation of Dicer1 in post-mitotic post-natal motor neurons fails to coordinate neurofilament subunit stoichiometry, but only the expression levels of NEFH were perturbed. Prediction analyses found one and nine binding sites for miR-9 in the 3′UTR of NEFL and NEFH, respectively. Direct inhibition of NEFH by miR-9 was confirmed by *in vitro* experiments (Table 1), but no luciferase assays were performed to validate the NEFL/miR-9 interaction. Thus, dysregulation of neurofilament stoichiometry in several motoneuron diseases is due to miR-9 loss (Haramati et al., 2010). No further efforts have been made to understand the involvement of miR-9 in ALS.

#### miRNA profile of the spinal cord in ALS

The first study aiming to characterize the miRNA profile in the spinal cord of sporadic ALS patients was conducted by Campos-Melo et al. (2013). They used a quantitative qRT-PCR-based array method to screen 664 human miRNAs from the spinal cords of three healthy controls and five ALS patients; they identified 246 down-regulated and 10 up-regulated miRNAs (Table 2). This was the only study reporting such a mass decrease in the miRNA profile for NDDs. Interestingly, many of the de-regulated miRNAs were predicted to have a binding site in the 3′UTR of NEFL, and consistent inhibition was demonstrated for miR-146^*^, miR-524-5p, and miR-582-3p (Campos-Melo et al., 2013). Around the same time, Koval et al. characterized the expression of 613 miRNAs using miRNA microarray experiments and the spinal cords of diseased rats and mice. Using individual assays, 11 miRNAs were confirmed in the diseased mice, 10 in SOD1^G93*A*^ rats, and 6 in ALS patients (miR-24-2^*^, miR-142-3p, miR-142-5p, miR-1461, miR-146b, and miR-155) (Table 2). More importantly, miR-155 was increased in both sporadic and familial ALS patients, and when its expression was inhibited in the brain of SOD1^G93*A*^ rats *in vivo*, both survival and disease duration were increased (Koval et al., 2013).

#### miRNAs in the peripheral tissues of ALS patients

De Felice et al. performed the first and only miRNA profiling of leukocytes isolated from blood to identify characteristic patterns in sporadic ALS patients (De Felice et al., 2012). Briefly, leukocytes were isolated from the blood of 8 patients and 12 healthy controls and screened for the expression of 911 human miRNAs using microarray technology. Eight miRNAs (miR-338-3p, miR-451, miR-1275, miR-328, miR-638, miR-149, miR-665, and miR-583) were de-regulated in ALS patients (Table 2). Among these miRNAs, miR-338-3p was previously found in brain tissue from ALS patients (Shioya et al., 2010). This study detected, for the first time, specific disease-related changes in miRNAs at an earlier stage of sporadic ALS.

Another study was performed on peripheral tissues from ALS patients; in particular, the analyses were restricted to a subgroup of monocytes (CD14^+^CD16^−^) isolated from ALS patients. This population was chosen because its murine analog (Ly6^Chi^monocytes) isolated from SOD1 mice has a pronounced pro-inflammatory profile (gene and miRNA expression) prior to disease onset and is recruited to the spinal cord, where the cells proliferate during disease progression. The human CD14^+^CD16^−^monocytes isolated from ALS patients and Ly6^Chi^ monocytes isolated from SOD1 mice had a unique inflammatory miRNA profile. Ly6^Chi^ diseased monocytes were characterized by the up-regulation of let- 7, miR-15b, miR-16, miR-27a, miR-34a, miR-132, miR-146a, miR-155, miR-223, and miR-451 (Table 2). Human CD14^+^CD16^−^ALS monocytes had higher expression levels of miR-27a, miR-155, miR-146a, and miR-32-3p (Butovsky et al., 2012) (Table 2). Finally, the authors underlined the potential role of these miRNAs as biomarkers of ALS.

### miRNAs and Huntington's disease (HD)

HD is an incurable neurodegenerative condition caused by CAG repeat expansion in the huntingtin gene (Htt). HD patients manifest cognitive defects and motor control impairment due to neuronal dysfunction characterized by progressive loss of cortical and striatal neurons. Neuronal death happens due to the toxicity associated with the mutant Htt protein and loss of the neuroprotective effects of the wild-type protein (Cattaneo et al., 2005). Little is known about the function of Htt, but its mutant form affects cellular phenotype and viability (Zuccato et al., 2010). Several genes have been found to be altered in the brain of HD patients (Cha, 2007), and many transcription factors (TFs) interact with Htt and are recruited to the mutant Htt aggregates (Sugars and Rubinsztein, 2003) in the brain. Recruitment to Htt aggregates prevents TFs from binding to DNA and eliciting their functions. More importantly, mutant Htt inhibits the formation of processing bodies (P bodies) by interacting with Ago1 and Ago2, which are involved in miRNA biogenesis (Savas et al., 2008). Thus, miRNA dysregulation is expected in the brain of HD patients. Currently, no cure exists for HD; all of the treatments are palliative. RNA therapy has emerged as a powerful tool for modifying the disease course by targeting mutant HTT mRNA for degradation.

#### miRNA profile in the brain of HD patients

Htt was demonstrated to interact with repressor element 1 silencing transcription factor (REST), the essential transcriptional repressor also known as neuron-restrictive silencing factor (NRSF), in neurons (Zuccato et al., 2003; Ooi and Wood, 2007). In control individuals, Htt sequesters REST in the cytoplasm of neurons and prevents the repressor from binding to DNA; in HD patients the mutant Htt does not associate with REST, which relocates to the nucleus of HD neurons and represses many of its target genes. One of the target genes of REST is BDNF, which is essential for neuron survival (Zuccato et al., 2003).

Based on the presence of REST binding sites in the genome, Johnson et al. were able to identify a set of REST-target miRNAs in the human genome (miR-9-1, 9-3, 29a, 29b-1, 124a-1, 124a-2, 124a-3, 132, 135b, 139, 203, 204, 212, 330, and 346) that are also brain or neuron-specific (Johnson et al., 2008). Among these miRNAs, four (miR-29a, miR-124a, miR-132, and miR-330) were found to be decreased in the cortex of R6/2 mice, an animal model of HD. Furthermore, their known target mRNAs were increased in R6/2 mice (Johnson et al., 2008) Johnson et al. then analyzed the expression profile of miR-29a, miR-124a, miR-132, and miR-135b in parietal cortical tissues from control and HD individuals; only down-regulation of miR-132 was confirmed in the human samples (Table 2). Otherwise, the expression levels of miR-29a and miR-330 were increased and miR-124a did not differ between HD and control subjects (Johnson et al., 2008) (Table 2).

Packer et al. investigated whether miRNAs correlate with disease progression in HD patients by analyzing the expression profile of predicted REST-regulated miRNAs in Brodmann's area 4 (BA4) isolated from control and HD grade 1–4 brain samples (Packer et al., 2008). Five miRNAs (miR-9, miR-9^*^, miR-29b, miR-124a, and miR-132) were significantly different with increasing HD grade (Packer et al., 2008). Otherwise, no correlation with disease progression was observed for miR-139, miR-135b, and miR-212. Next, a qRT-PCR-based miRNA array platform was used to evaluate the expression profiles of 365 mature miRNAs in the BA4 cortex from control and early stage HD (grades 1 and 2) patients. The de-regulation of additional miRNAs, including miR-486, miR-196a, miR-17-3p, miR-22, miR-485-5p, miR-500, and miR-222, was found (Packer et al., 2008).

Finally, Marti et al. analyzed the expression profile of miRNAs in the frontal cortex and striatum of HD patients using three different techniques: RNA sequencing, microarray, and qRT-PCR. miR-100, miR-151-3p, miR-16, miR-219-2-3p, miR-27b, miR-451, and miR-92a were found to be over-expressed in diseased tissues in all three experiments (Marti et al., 2010). Similarly, miR-128, miR-139-3p, miR-222, miR-382, miR-433, and miR-483-3p were decreased in the HD brain tissue in all three experiments (Marti et al., 2010).

Based on the data above, 30 miRNAs are increased and 24 miRNAs are decreased in the brains of HD patients. De-regulation of 33 of the 54 miRNAs associated with HD can be attributed to four TFs that are altered in the HD brain; TP53, REST, E2F1, and GATA4 (Sinha et al., 2012). In particular, TP53 is involved in processing the primary miRNA transcript to the mature miRNA (Suzuki et al., 2009). Because intronic miRNAs are transcribed at the same levels as the host genes if oriented in the same direction (Baskerville and Bartel, 2005; Wang et al., 2009), Sinha et al. investigated a possible relationship between the host genes and intronic miRNAs in HD. Thirty-one of the 54 miRNAs de-regulated in the brains of HD patients are encoded within the introns, and the expression of some of these miRNAs correlates with the expression levels of their host genes (Sinha et al., 2012).

#### miR-9/9^*^-REST/CoREST feedback in HD

As mentioned above, the expression levels of miR-9 and miR9^*^ are decreased in the cerebral cortex of HD-affected subjects. Interestingly these two miRNAs target REST and CoREST, respectively (Johnson and Bucley, 2009a). REST has been demonstrated to inhibit the expression of neuronal genes in non-neuronal cells, and under normal conditions it is retained in the cytoplasm by interacting with Htt. When Htt is mutated, REST no longer associates with Htt, which then relocates and accumulates in the nucleus, where it inhibits the expression of several genes (Zuccato et al., 2003). Decreased levels of miR-9/9^*^ in HD would increase the transcription of REST, amplifying the accumulation of this protein in the presence of mutated Htt. This phenomenon is further magnified because miR-9/9^*^ transcription depends on REST. Thus, the translocation of REST to the nucleus in HD brain tissues explains the reduced expression of miR-9/miR9^*^ (Johnson et al., 2009b).

#### miR-196a and Htt expression

Based on the results published by Packer et al. (2008) and on unpublished microarray data from transgenic monkeys with HD, Cheng et al. identified miR-196a as a possible miRNA involved in the pathogenesis of HD. To further investigate the role of this miRNA in HD, they co-transfected human embryonic kidney and murine neuroblastoma cell lines with two constructs: miR-196a mimic and the mutant form of Htt. These *in vitro* experiments showed for the first time that miR-196a suppresses the expression of mutant Htt at the mRNA and protein levels. Furthermore, this inhibition was not due to the direct binding of miR-196a to the3′UTR of mutated Htt. Otherwise, miR-196a predominantly suppressed Htt expression through the inhibition of protein synthesis and partly through enhanced protein degradation. To confirm these results *in vivo*, Cheng et al. generated a miR-196a transgenic mouse and bred it with transgenic mice expressing mutant Htt fused to green fluorescent protein (GFP). The expression of miR-196a and mutant Htt were up- and down-regulated, respectively, in the brain of double transgenic mice, confirming the *in vitro* results. Moreover, inhibition by miR-196a also occurred at later stages of the disease in double transgenic mice when more Htt aggregates accumulated. Because the double transgenic mouse model represents a model of over-expression, Chen et al. evaluated the inhibition of mutated Htt via miR-196a by transfecting induced pluripotent stem cells derived from individuals with HD (HD-iPSCs) with lentiviral vector encoding for miR-196a. As expected, untreated cells accumulated more mutated Htt aggregates, whereas cells transfected with miR-196a were characterized by lower expression of mutated Htt, suggesting that miR-196a can alleviate the pathological phenotypes in human samples. The downstream effect of miR-196a over-expression on Htt metabolism was investigated; the ubiquitin-proteosome system, gliosis, cAMP response element-binding protein pathway, and several neuronal regulatory pathways were implicated (Cheng et al., 2013). All of these evidences suggest a potential therapeutic role of miR-196a in HD.

#### miRNAs in the peripheral tissues of HD patients

Even though the ultimate trait biomarker is represented by mutated Htt, many efforts have focused on identifying mRNAs or proteins with expression profiles that could correlate with disease progression. Gaughwin et al. developed a cell model of HD (HTT-Exon-1 over-expressing human cell line) in order to identify miRNA biomarkers. Briefly, they transfected an embryonal carcinoma-derived pluripotent cell line (NT2) capable of differentiating into neurons with Htt-Exon-1 construct carrying 23, 73, and 145 polyglutamine repeats. Microarray analysis revealed two known miRNAs (miR-34b and miR-1285) that are increased in the presence of 73Q-Htt and 145Q-Htt compared to23Q-Htt. Based on these data, they investigated the expression levels of miR-34b and miR-1285 in human plasma, demonstrating that they are detectable in human samples and bio-stable relative to proteins. When the investigation was expanded to plasma from HD patients, miR-34b was increased in pre-manifest HD plasma relative to age-matched controls (Gaughwin et al., 2011). In contrast, no correlation was found for miR-1285. These results suggest that miR-34b behaves as a potential biomarker of HD prior to symptom onset. Despite the novelty of the results obtained, a limitation of this study was the small patient cohort, which needs to be enlarged.

## Therapeutic miRNAs for NDDs

The essential properties of a drug are favorable bioavailability, a reasonable half-life, and few side effects. These requirements are dependent on the type of drug, the target organ, and on the delivery system used. An ideal vector for *in vivo* delivery of RNA molecules should be equipped with a cationic group for effective transfection, an endosomolytic group for endosomal escape, a surface modifier to decrease steric hindrance, which enhances circulation in the blood, and a targeting moiety to direct the delivery system at target cells or tissue (Whitehead et al., 2009). From the injection of miRNA agonists/antagonists and knockdown of target genes/endogenous miRNAs, physiological barriers represent the first obstacle to the efficacy of drug treatment. Many checkpoints are represented by glomerular filtration, hepatic metabolism, reticular endothelial system (RES) uptake, and endothelial barriers. If injected as naked molecules, RNA is subjected to nuclease degradation, which is responsible for 70% knockdown of drug efficacy within 1 min of administration (Mahato et al., 1995). To avoid the action of nucleases, chemical modification or non-viral carriers can be used (Borchard, 2001; Wang et al., 2002; Crooke, 2004; Juliano, 2005; Juliano et al., 2008). RNA particles >200 nm delivered to liposomes, lipoplexes, polyplexes, or nanoparticles are subjected to phagocytosis by RES (Alexis et al., 2008), and those smaller than 100 nm are the target of hepatic Kupffer cells. Conjugation with non-viral carriers might induce marked toxicity because RNA molecules will also enter the non-targeted cells due to an interaction between the negatively charged cellular membrane and cationic carriers (Uyechi et al., 2001). This effect might be reduced by coating carriers with hydrophilic molecules (polyethylene glycol) or by conjugation with a ligand (e.g., surface receptor-specific antibodies) (Balyasnikova et al., 2002). Furthermore, RNA molecules can be associated with aptamers, monoclonal antibodies, or peptides to target specific cell surface receptors and the desired target in the body (Juliano, 2005; Chu et al., 2006; Kumar et al., 2008). After RNA molecules have passed these physiological barriers, they have to enter the target cells to elicit their actions. This means that they have to cross the cell membrane, escape endosomes, and localize in the nucleus. Therefore, nuclear-localization signals and cell-penetrating and endosomal-release signal peptides can influence the duration of action of injected RNA molecules (Jere et al., 2009).

**Figure 1:**
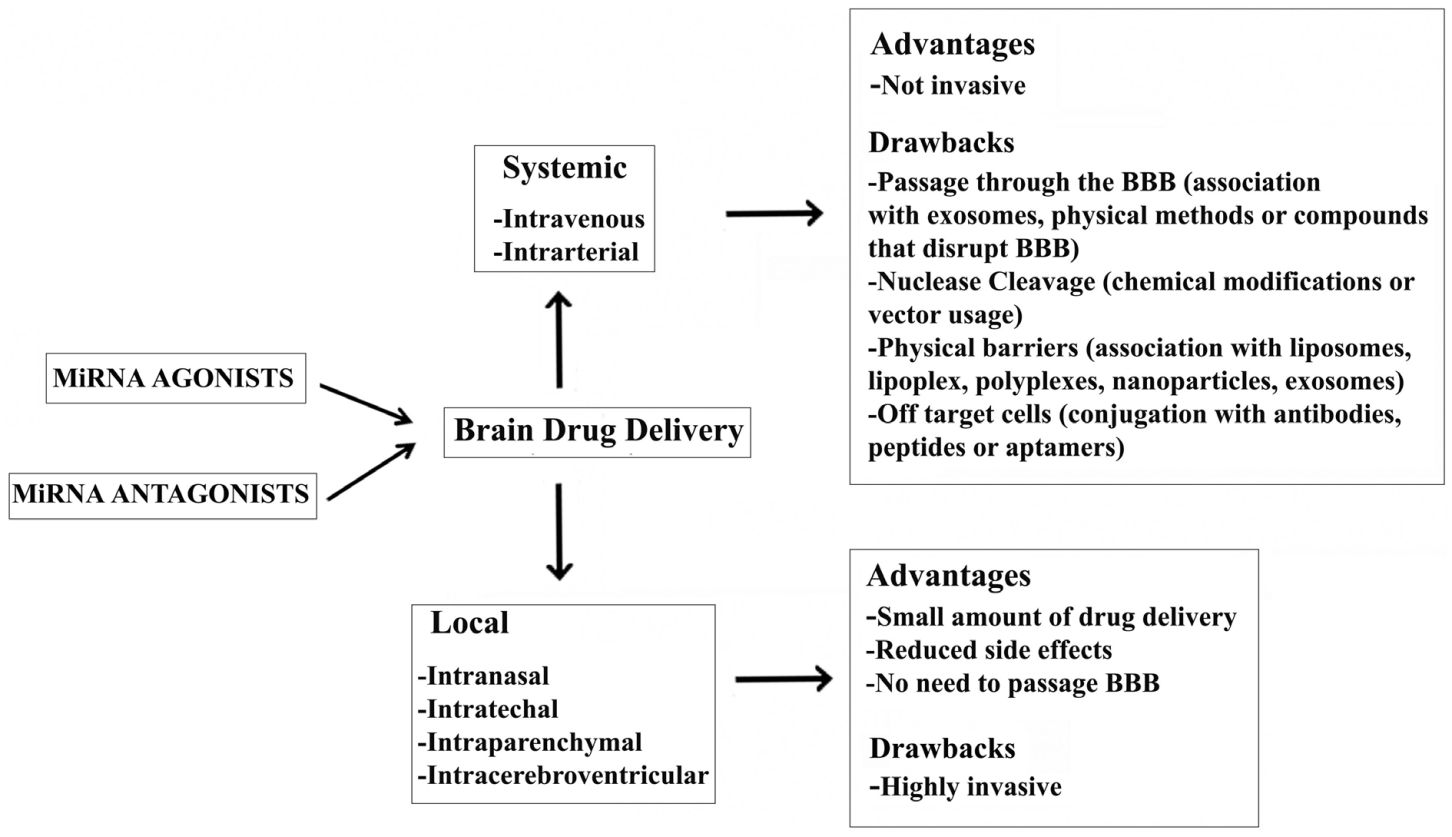
**Advantages and drawbacks of a miRNA-based therapy for the treatment of NDDs**.

*In vivo* delivery of RNA can be achieved two ways: systematically and locally (Figure 1). A great amount of drug is required when it is injected systemically. In contrast, local delivery allows a small amount of drug to be administered with reduced side effects (Pardridge, 2007; Pushparaj et al., 2008; Shen, 2008). Notably, systemic delivery is preferred when the target organ is the liver as the majority of systemically administered drug molecules localize to the liver. Efforts have been made to deliver RNA molecules across the BBB by *in vivo* systemic delivery, but it remains a major challenge in the treatment of NDDs. In particular, physical methods, such as ultrasound (Chen et al., 2010; Liu et al., 2010), and the intra-arterial infusion of compounds that disrupt the BBB (e.g., potassium channel agonists and minoxidil sulphate) increase the chances of overcoming the BBB (Ningaraj et al., 2007; Bidros et al., 2010). Recently, Alvarez-Erviti et al. experimented the delivery of RNA molecules associated with exosomes across the BBB. Exosomes are cell-derived vesicles that enable cell-to-cell communication by transferring RNA molecules and proteins. They have been shown to preserve mRNAs and miRNAs in the presence of RNase and subsequently deliver them to recipient cells (Valadi et al., 2007; Skog et al., 2008; Zomer et al., 2010). In particular, Alvarez-Erviti et al. isolated brain-targeting exosomes from dendritic cells bioengineered to express an exosomal membrane protein (Lamp 2b) fused to a ligand of the acetylcholine receptor. Exosomes were then loaded with siRNAs targeting BACE1 mRNA by electroporation and injected intravenously, resulting in a significant knock-down of BACE1 expression (Alvarez-Erviti et al., 2011).

Efficient local delivery strategies for the CNS are intranasal, intracerebroventricular, intrathechal, or intraparenchymal injection of naked RNAi formulated in isotonic saline buffer (Makimura et al., 2002). Adenoviral, lentiviral, and adeno-associated virus-based local delivery has also been performed in animal models of AD, HD (Harper et al., 2005), and ALS (Ralph et al., 2005), demonstrating significant improvement. Nevertheless, the strategy to locally deliver a drug to the brain is still far from normal practice because of the complexities associated with direct injection into the brain.

## Conclusion

Following the discovery of miRNAs, their actions were investigated in almost all biological processes and, even more importantly, their central role in gene-expression regulation implicated in many human diseases (Subramanian et al., 2008; Thum et al., 2008; Eisenberg et al., 2009; Malumbres et al., 2009; Matkovich et al., 2009; Crist and Buckingham, 2010; Maciotta et al., 2012). miRNAs are of particular interest in understanding complex disorders, such as NDDs, because they can potentially regulate several pathways involved in the insurgence and progression of the disease. In the last few years, miRNAs have also been considered as biomarkers; they offer several advantages over mRNA or protein, including increased stability and biological relevance in many different diseases. In fact, miRNAs offer the possibility to link a biomarker with an altered biological process and therapy capable of targeting the pathological mechanism. miRNA-based therapeutic treatments for NDDs may follow two different strategies: miRNA over-expression (gain-of-function) or miRNA repression (loss-of-function) (Figure 1). The first approach might use miRNA-associated target gene specificity in order to down-regulate the expression of the aberrant gene within the cell of interest; the second should use miRNAs to directly influence the differentiation of neural stem cells (NSCs) (Palm et al., 2012).

In the last few years, the hypothesis that miRNAs could be involved in NDDs has gained support (Hebert and De Strooper, 2007) due to many experiments with different animal models, such as the fly and mouse. Much experimental data demonstrate that the miRNA network is necessary for neuron survival (Hebert and De Strooper, 2009). Experiments conducted in humans support the idea that changes in miRNA expression profiles or miRNA targets could increase the risk of major NDDs, such as AD and PD (Tables 1, 2).

miRNA research seems particularly promising for understanding not only the very prevalent and poorly understood sporadic forms of AD, but also forms of PD. The challenge now is to understand the role of specific miRNAs in biological models and translate this knowledge to clinical studies (Hebert and De Strooper, 2009).

The use of miRNAs as potential therapeutic targets remains controversial with regard to methods of delivery and target specificity. When considering a treatment for NDDs mediated by miRNA delivery, we have to evaluate its ability to pass through the BBB. In order to overcome the problem of the BBB, several siRNA delivery systems are being developed for *in vivo* purposes, including vector-based, chemically modified, and “packaged” RNA oligonucleotides (Kim and Rossi, 2007). Progress in the latter area will immediately translate into progress in the miRNA area because both are based on the same principles. Both small RNAs regulate at the post-transcriptional level; therefore, miRNAs and siRNAs are chemically identical. However, the big question is whether these different approaches will result in clinically feasible therapies because of bioavailability and toxicity issues inherent to all of these approaches, and the BBB constitutes an enormous hurdle for the effective delivery of these experimental drugs in the brain.

Oligonucleotides can be effectively deployed in animal models, and RNA complexity provides the opportunity to uncover novel regulatory mechanisms and biomarkers. A limit to clinical miRNA use is that much remains to be improved in the prediction of target genes for both miRNAs and lncRNAs (Johnson et al., 2012). The most effective way of interfering with ncRNA action is likely not by targeting the RNA/target gene interaction itself, but to target the recruited epigenetic apparatus; this offers the advantage of exploiting a growing array of chemical compounds aimed at the active sites of chromatin modifiers (Kelly et al., 2010). At the very least, this expanded view of the importance of RNA, both protein-coding and non-coding, both small and large, offers an abundance of novel interactions to target that are distinct from the current focus on protein regulation in neurodegeneration.

Small ncRNAs add a novel and exciting layer of complexity to molecular neuronal biology. In addition, publications will exponentially increase in the years to come, which will provide novel insights into this recently discovered field of research (Hebert and De Strooper, 2009). A “many to many” relationship exists between miRNAs and their target mRNAs. The ability of a single miRNA to potentially target as many as 200 different miRNAs is well documented, but there is also evidence of single mRNA being targets of multiple miRNAs. To put this complex “many to many” relationship in a biological context, a comprehensive analysis of all miRNA targets suggested to be regulated by a single miRNA generally constitutes a biological network of functionally associated molecules in human cells. This evidence may represent a limitation for the use of regulatory small RNAs as a biomarker in NDDs or for future clinical trials to treat NDDs. However, miRNAs might help extract some biologically relevant targets among the high number of “predicted targets” of individual miRNAs, and potentially serves as a filter when using pathway analysis tools to understand the functional pathways affected by miRNA profile changes in NDDs.

In conclusion, many scientific questions remain to be addressed before efficient delivery and/or modulation of miRNAs in the brain will be possible (Krutzfeldt et al., 2005, 2007).

### Conflict of interest statement

The authors declare that the research was conducted in the absence of any commercial or financial relationships that could be construed as a potential conflict of interest.
